# Cerebrospinal Fluid Levels of Autophagy-related Proteins Represent Potentially Novel Biomarkers of Early-Stage Parkinson’s Disease

**DOI:** 10.1038/s41598-018-35376-6

**Published:** 2018-11-15

**Authors:** Jinyoung Youn, Sang-Bin Lee, Hyo Sang Lee, Hyun Ok Yang, Jinse Park, Ji Sun Kim, Eungseok Oh, Suyeon Park, Wooyoung Jang

**Affiliations:** 1 Department of Neurology, Samsung Medical Center, Sungkyunkwan University School of Medicine, Seoul, Republic of Korea; 2 Neuroscience Center, Samsung Medical Center, Seoul, Republic of Korea; 3 Natural Products Research Center, Korea Institute of Science and Technology, Gangneung, Republic of Korea; 4 School of Pharmacy, Sungkyunkwan University, Suwon, Republic of Korea; 5 Department of Nuclear Medicine, Gangneung Asan Hospital, University of Ulsan College of Medicine, Gangneung, Republic of Korea; 6 Department of Neurology, Inje University, Haeundae Paik Hospital, Busan, Republic of Korea; 7 Department of Neurology, Chungnam National University College of Medicine, Chungnam National University Hospital, Daejeon, Republic of Korea; 8 Department of Biostatistics, Soonchunhyang University Hospital, Seoul, Republic of Korea; 9 Department of Neurology, Gangneung Asan Hospital, University of Ulsan College of Medicine, Gangneung, Republic of Korea; 10 Division of Bio-medical Science and Technology, KIST School, Korea University of Science and Technology, Seoul, Republic of Korea

## Abstract

The roles of autophagy-related proteins as diagnostic or monitoring biomarkers in Parkinson’s disease (PD) have not been clearly elucidated. We recruited 32 patients with early-stage PD and 28 control subjects, and evaluated parkinsonian motor symptoms and dopamine transporter imaging data. Cerebrospinal fluid (CSF) levels of LC3B, Beclin1, and LAMP-2 were estimated using ELISAs, and CSF levels of ATG5, ATG7, and p62 were examined by immunoblotting. Additionally, we also assessed the levels of α-synuclein, total tau, and phosphorylated tau in CSF using ELISAs. Significant differences in the levels of LC3B, LAMP-2, and Beclin1 were observed between the PD and control groups. Using 29.8 pg/mL as the cut-off value for a diagnostic biomarker of PD, CSF LC3B levels exhibited high sensitivity (96.9%) and specificity (89.3%) with an area under the curve of 0.982. Furthermore, LC3B was significantly correlated with the asymmetry index in the caudate and putamen, as estimated by a semi-quantitative analysis of [^18^F] *N*-(3-fluoropropyl)-2β-carbon ethoxy-3β-(4-iodophenyl) nortropane (FP-CIT) positron emission tomography (PET). CSF levels of LC3B represented a potential diagnostic and prognostic biomarker of early-stage PD in patients. Based on our findings, molecular biological changes in PD are associated with dysregulation of the lysosomal autophagy pathway.

## Introduction

Parkinson’s disease (PD) is a common and disabling neurodegenerative disease characterized by various motor and non-motor symptoms^1^. However, all current treatment modalities are only designed to manage symptoms by stimulating the dopaminergic system, and not modify the underlying disease in patients with PD^2^. The first step in the development of disease-modifying strategies is to identify ideal biomarkers for early diagnosis and monitoring disease progression^3^; thus, suitable biomarkers of PD in patients are urgently needed^3,4^.

The effective biomarkers of PD should be based on the pathogenic processes observed in patients with PD^4^. In this respect, the cerebrospinal fluid (CSF) is a potentially reliable source of biomarkers of PD, because peptides or proteins in the CSF might directly reflect the pathological changes or brain-specific activities in neurodegenerative process^5^. Although biomarkers derived from diverse mechanisms, including free radicals, mitochondrial dysfunction, excitotoxicity, and inflammation, have been proposed for PD, many studies have reported the critical role of abnormal misfolded proteins, including α-synuclein, tau, and amyloid-β, in PD^6,7^. However, a consensus regarding the use of the aforementioned proteins as biomarkers in patients with PD is still lacking^8–10^. In terms of α-synuclein levels in CSF, some studies, but not others, have reported decreased levels of α-synuclein in patients with PD^11^. Furthermore, a large overlap in the CSF α-synuclein level was observed between patients with PD and patients with other neurodegenerative diseases^12^. Similarly, conflicting results for other misfolded proteins have been reported in previous studies^12–14^.

Among the various suggested mechanisms of PD, dysregulation of the lysosomal autophagy pathway has been observed in patients with PD and animal models^15^. Moreover, lysosomal activity is required for α-synuclein degradation, and the activation of autophagy reduces toxic aggregates in PD models^16,17^. Recently, several studies have examined the GBA gene encoding β-glucocerebrosidase (GCase), which is related to autophagy function^18^. Reduced GCase activity has been observed in post-mortem brain tissues and CSF from patients with PD, and GCase activity was evaluated as a biomarker for differentiating PD from neurological controls^18^. Therefore, CSF biomarkers reflecting the lysosomal autophagy pathway could be useful for patients with PD.

However, few investigations have directly measured the levels of autophagy-related (ATG) proteins as biomarkers of PD, and only one study reported the differential expression of specific ATG proteins in patients with PD^19^. However, in that study, the sample size was very small and only limited numbers of ATG proteins were evaluated. Moreover, the authors enrolled patients with PD, regardless of disease stage, and focused on the roles of biomarkers in differentiating between PD and atypical parkinsonism, not on the use of biomarker for early diagnosis or monitoring disease severity.

Therefore, we compared Beclin1, ATG5, ATG9, L3CB, p62, and LAMP-2, levels between patients with PD and normal controls, and investigated their correlations with clinical severity, quantitative dopamine transporter imaging data, and conventional CSF biomarkers in patients with PD to investigate the roles of various ATG proteins as biomarkers of the pathogenic process in patients with PD.

## Results

### Basic demographic and clinical characteristics of study subjects

The demographic and clinical characteristics of all enrolled subjects are presented in Table 1. Significant differences in age, gender, MoCA-K and BDI scores, and routine serum laboratory parameters were not observed between patients with PD and the control group. In a semi-quantitative analysis of [^18^F] *N*-(3-fluoropropyl)-2β-carbon ethoxy-3β-(4-iodophenyl) nortropane (FP-CIT) positron emission tomography (PET), significantly lower uptake of the radiolabel was observed in both the putamen and caudate of patients with PD than in control group. The asymmetry index (AI) of the caudate and putamen in the PD group was also significantly increased compared with the control group (Table 1).

**Table 1:** The baseline demographic, clinical, and laboratory parameters of the enrolled subjects.

	PD (n = 32)		Control (n = 28)		*p*-value
	Mean	SD	Mean	SD	
Age	64.31	8.49	66.18	12.75	NS
Gender (male/female)	13/19		8/20		NS
Disease duration (month)	13.03	10.63			
UPDRS part 3	22.66	6.20			
Modified H &Y	1.59	0.53			
PD Subtypes (TD/Mixed/AR)	11/16/6				
NMSS	28.84	15.60			
BDI	7.06	6.29	5.86	7.08	NS
MoCA-K	25.03	1.94	25.64	2.02	NS
Lab parameters (serum)					
Hb/Haematocrit	12.93/39.46	1.79/4.43	13.16/39.45	1.19/3.34	NS/NS
BUN/Creatinine	14.96/0.95	3.89/0.19	15.05/0.90	4.12/0.15	NS/NS
AST/ALT	21.28/21.56	7.40/14.37	22.74/18.42	7.10/10.92	NS/NS
Lab parameters (CSF)					
Alpha-synuclein (pg/mL)	116.76	34.25	176.60	50.61	<**0.01**
Total Tau (pg/mL)	76.87	33.53	104.71	47.72	<**0.05**
Phosphorylated tau (pg/mL)	8.80	5.46	9.81	8.55	NS
Tau ratio	0.18	0.14	0.14	0.16	NS
FP-CIT PET analysis					
Left caudate	3.48	0.94	5.58	0.85	<**0.01**
Left putamen	3.73	0.99	4.57	1.06	<**0.01**
Right caudate	3.48	1.05	4.69	0.92	<**0.01**
Right putamen	3.51	1.08	4.57	1.06	<**0.01**
AI, caudate	7.67	7.47	2.87	6.68	<**0.05**
AI, putamen	13.75	13.81	3.02	2.44	<**0.01**

PD: Parkinson’s disease; SD: standard deviation; UPDRS: Unified Parkinson’s Disease Rating Scale; H &Y: Hoehn and Yahr stage; TD: tremor-dominant; AR: akinetic-rigid; NMSS: Non-motor Symptom Scale; BDI: Beck Depression Inventory; MoCA-K: The Korean version of Montreal Cognitive Assessment; Hb: hemoglobin; BUN: blood urea nitrogen; AST: aspartate aminotransferase; ALT: alanine aminotransferase; CSF: cerebrospinal fluid; NS: not significant; FP-CIT PET: [^18^F] *N*-(3-fluoropropyl)-2β-carbon ethoxy-3β-(4-iodophenyl) nortropane PET

Tau ratio is defined as the ratio of phosphorylated tau to total tau.

The AI was calculated as follows: (better uptake - worse uptake)/better uptake * 100.

*Adjusted for age.

Bold: statistically significant difference.

### Comparison of CSF biomarkers between the PD and control groups

**Figure 1:**
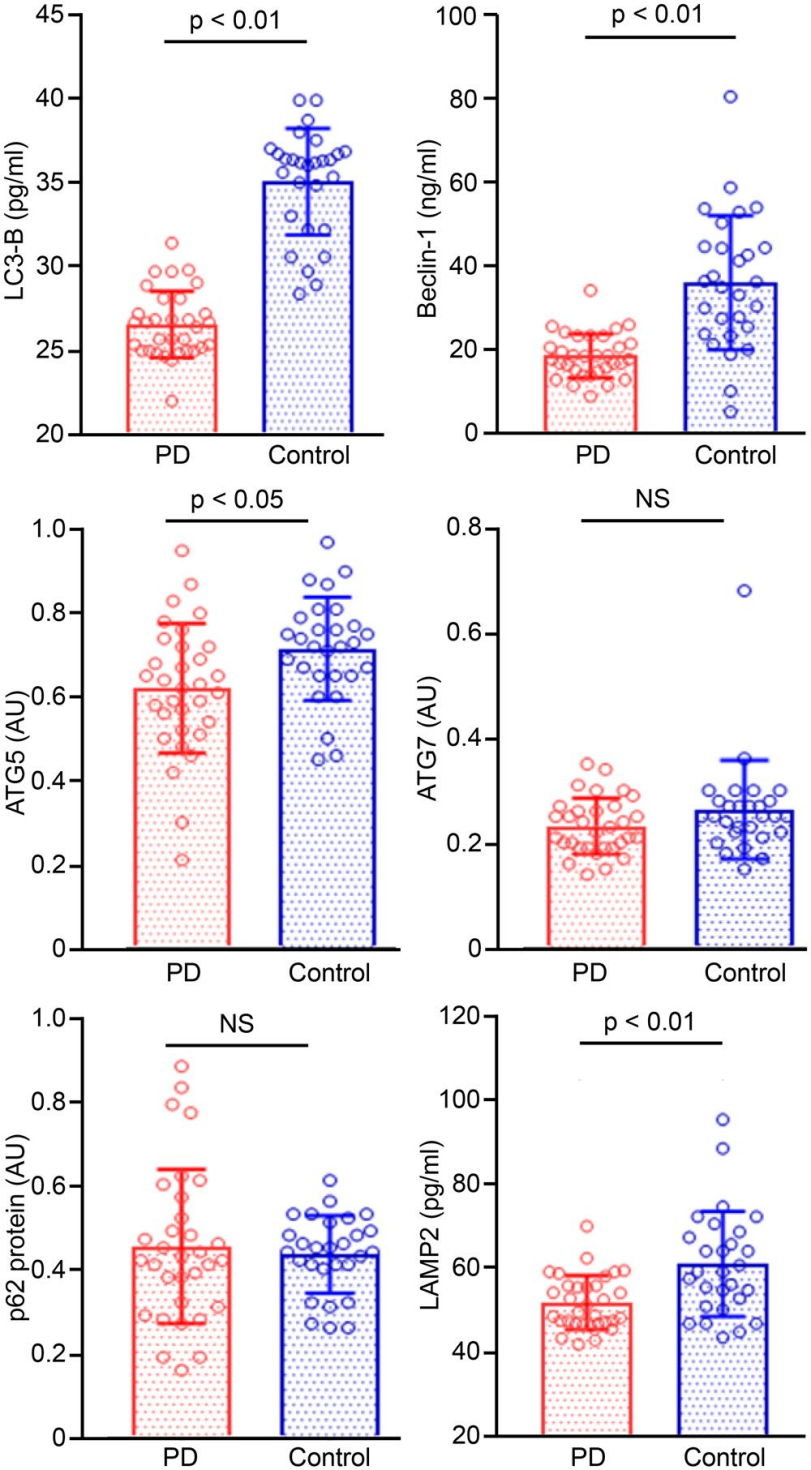
Comparison of the CSF levels of autophagy-related proteins between the PD and control groups. LC3B, Beclin1, ATG5, and LAMP-2 levels were significantly decreased in patients with PD compared with normal controls.

Figure 1 shows a comparison of the levels of ATG proteins in the CSF between the PD group and control group. LC3B, Beclin1, and LAMP-2 were present at detectable levels in the CSF using ELISAs. Significantly lower levels of LC3B and Beclin1, which reflect macroautophagy, were observed in patients with PD than in the control group. In addition, LAMP-2 levels were also significantly reduced in patients with PD compared with the control group. When we compared the levels of ATG proteins using Western blotting, significantly lower levels of the ATG5 protein were detected in the PD group than in the control group, but to the levels of the p62 and ATG7 proteins were not significantly different between groups.

A comparison of the levels of other CSF biomarkers between the two groups showed significant differences in α-synuclein and total tau levels (Table 1). The α-synuclein level in the CSF from patients with PD was reduced compared with the control group, while a significantly higher total tau level was detected in patients with PD than in controls. In contrast, significant differences in the p-tau to tau ratio in the CSF were not observed between the two groups. Compared with previously established conventional CSF markers, only LC3B showed a significant correlation with other CSF markers (Table 2). The LC3B level correlated positively with α-synuclein and total tau levels, and negatively with the tau ratio.

**Table 2:** Correlations between the levels of autophagy-related proteins in the CSF and conventional CSF or clinical markers in patients with PD.

	LC3-B		Beclin-1		ATG5		LAMP-2	
	r	*p*-value	r	*p*-value	r	*p*-value	r	*p*-value
α-synuclein (CSF)	0.51	<**0.01**	−0.15	NS	0.17	NS	0.25	NS
Total tau (CSF)	0.51	<**0.01**	−0.27	NS	0.27	NS	0.20	NS
P-tau (CSF)	0.20	NS	0.30	NS	0.10	NS	−0.34	NS
Tau ratio (CSF)	−0.38	<**0.05**	0.16	NS	−0.07	NS	−0.29	NS

PD: Parkinson’s disease; UPDRS: Unified Parkinson’s Disease Rating Scale; NMSS: Non-motor Symptom Scale; NS: not significant.

**Figure 2:**
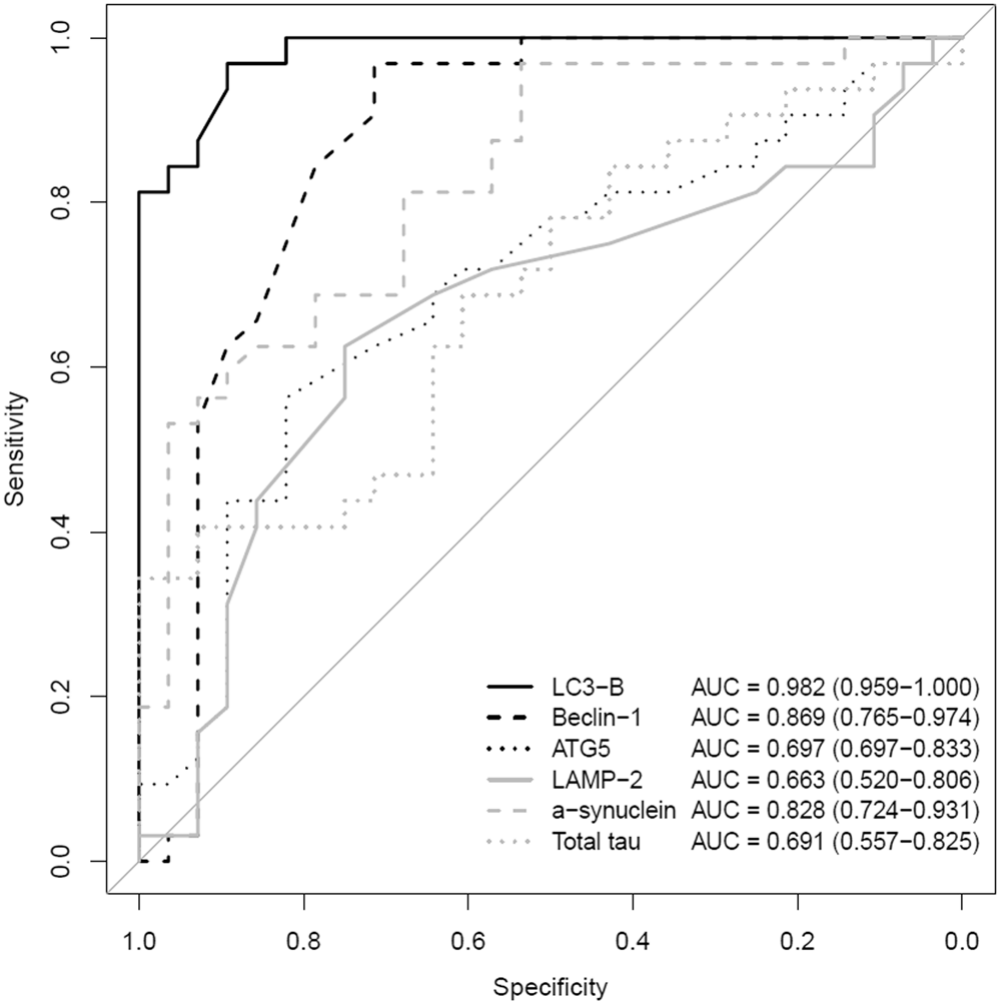
ROC curve showing the diagnostic performance of CSF biomarkers. With 29.8 pg/mL as the cut-off value for the differential diagnosis of PD from normal controls, the CSF level of LC3B exhibited high sensitivity (96.9%) and specificity (89.3%), with an AUC of 0.982 (0.959−1.000). ROC, receiver operating characteristic; PD, Parkinson’s disease.

The sensitivity and specificity of the levels of CSF biomarkers for the diagnosis of PD are shown in Fig. 2. Among the various CSF biomarkers studied here, the CSF LC3B level showed a high sensitivity (96.9%, 95% confidence interval (CI): 83.8–99.9%) and specificity (89.3%, 95% CI: 71.8–97.7%). The area under the curve (AUC) was 0.982 (0.959−1.000), with a cut-off value of 29.8 pg/mL. Additionally, the CSF Beclin1 and α-synuclein levels also displayed high sensitivity, but relatively low specificity (Table 3 and Fig. 2).

**Table 3:** Results of the discriminant analysis using the CSF biomarker levels for a differential diagnosis of PD from normal controls.

	Sensitivity (95% CI)	Specificity (95% CI)	AUC (95% CI)	cut-off value
LC3B	96.9% (83.8–99.9)	89.3% (71.8–97.7)	0.982 (0.959–1.000)	29.8 pg/mL
Beclin1	96.9% (83.8–99.9)	71.4% (51.3–86.8)	0.869 (0.765–0.974)	0.26 ng/mL
ATG5	56.3% (37.7–73.6)	82.1% (63.1–93.9)	0.697 (0.697–0.833)	0.64 AU
LAMP-2	62.5% (43.7–78.9)	75.0% (55.1–89.3)	0.663 (0.520–0.806)	0.31 pg/mL
α-synuclein	96.9% (83.8–99.9)	53.6% (33.9–72.5)	0.828 (0.724–0.931)	174.5 pg/mL
Total tau	34.4% (18.6–53.2)	100.0% (100.0–100.0)	0.691 (0.557–0.825)	38.57 pg/mL

CSF: cerebrospinal fluid; PD: Parkinson’s disease; CI: confidence interval.

### Correlations between the levels of autophagy-related proteins and clinical severity

When we analysed the correlations between the levels of ATG proteins and clinical severity in patients with PD, LC3B was the only ATG protein that showed a significant correlation (Table 4). LC3B was correlated with the severity of parkinsonian motor symptoms, but not with the severity of non-motor symptoms.

**Table 4:** Partial correlation analysis of clinical data and FP-CIT PET uptake values in relation to CSF autophagy-related proteins in patients with PD.

	LC3B		Beclin1		ATG5		LAMP-2	
	r	*p*-value	r	*p*-value	r	*p*-value	r	*p*-value
Clinical scale								
UPDRS part 3	−0.39	**<0.05**	−0.32	NS	−0.15	NS	0.04	NS
NMSS (total)	0.01	NS	0.06	NS	0.10	NS	0.14	NS
**SNBR**								
Right caudate	0.08	NS	−0.33	NS	0.01	NS	0.28	NS
Right putamen	0.06	NS	−0.28	NS	0.01	NS	0.31	NS
Left caudate	0.30	NS	−0.01	NS	0.32	NS	−0.10	NS
Left putamen	0.22	NS	0.04	NS	0.33	NS	−0.01	NS
AI (caudate)	−0.37	<**0.05**	0.21	NS	0.13	NS	−0.19	NS
AI (putamen)	−0.44	<**0.05**	−0.43	<**0.05**	0.31	NS	−0.09	NS

FP-CIT PET: [^18^F] *N*-(3-fluoropropyl)-2β-carbon ethoxy-3β-(4-iodophenyl) nortropane PET; CSF: cerebrospinal fluid; PD: Parkinson’s disease; UPDRS: Unified Parkinson’s Disease Rating Scale; NMSS: Non-motor Symptom Scale; SNBR: specific to non-specific binding ratio; AI: asymmetry index.

Adjusted for age.

Bold values indicate statistically significant differences; NS: not significant.

The AI was calculated as follows: (better uptake - worse uptake)/better uptake * 100.

**Figure 3:**
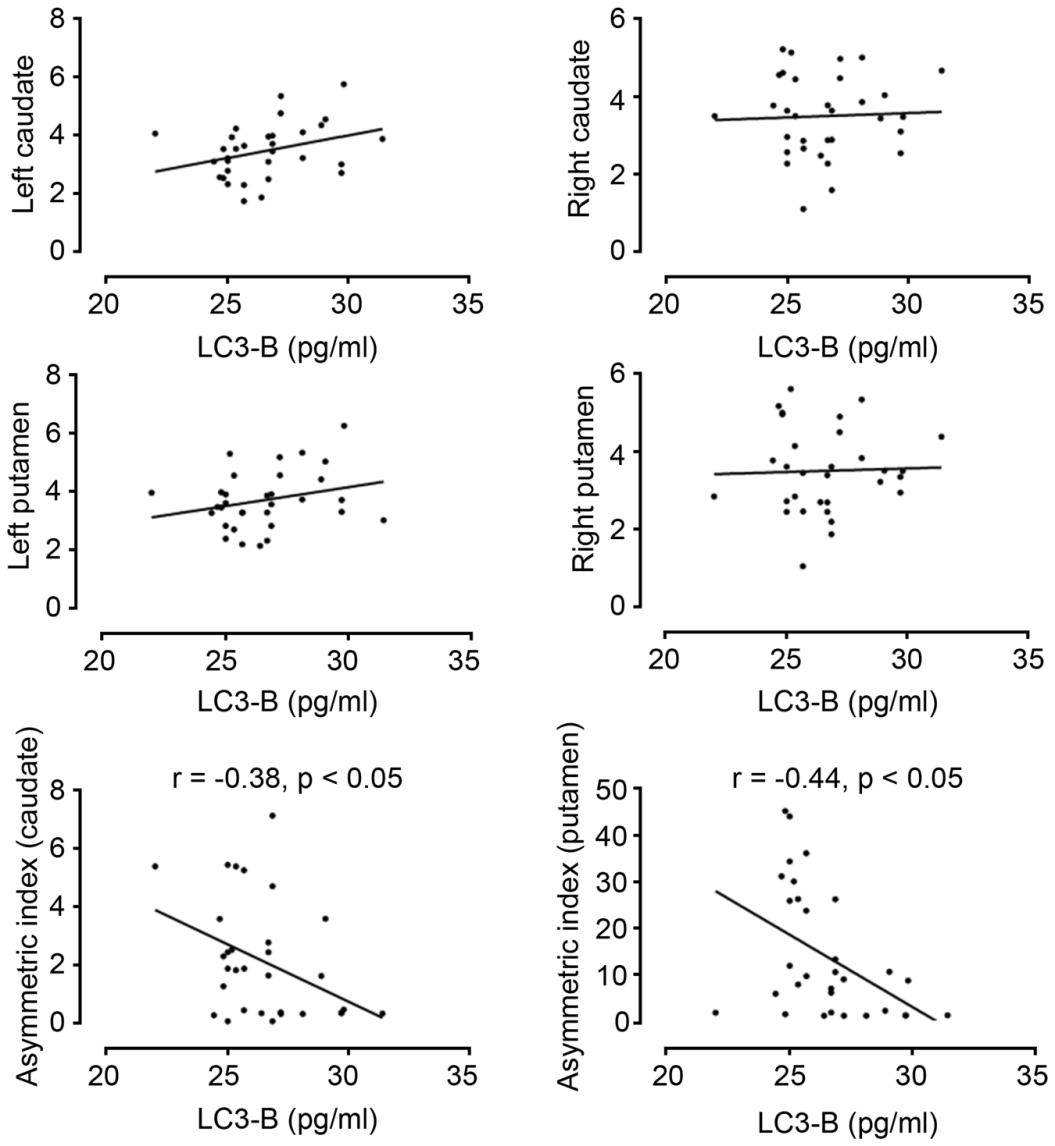
Correlation of LC3B level with quantitative FP-CIT PET data. Among the autophagy-related proteins, LC3B showed significant correlations with AIs in both the caudate and putamen, regardless of age.

In the analysis of the correlation between ATG proteins and FP-CIT PET data adjusted for age, LC3B showed a significant negative correlation with AIs in both the caudate and putamen (Table 4 and Fig. 3). Beclin-1 also showed a negative correlation with the AI in the putamen, but not with the AI in the caudate. Regarding conventional CSF markers, the total tau level was positively correlated with the specific to non-specific binding ratio (SNBR) in the left caudate and negatively correlated with the AI in the putamen (Supplementary Table). Additionally, α-synuclein and p-tau levels were positively correlated with the SNBR in the left putamen; however, significant correlations were not observed with the SNBR in other ROIs and AIs.

## Discussion

In the present study, we focused on ATG proteins as CSF biomarkers for patients with early-stage PD. We observed significant differences in CSF levels of LC3B, Beclin1, ATG5 and LAMP-2 between patients with early-stage PD and healthy controls. In particular, LC3B was well-correlated with the severity of motor symptoms, AIs in the caudate and putamen in a semi-quantitative analysis of dopamine transporter imaging, and some conventional CSF biomarkers, supporting the hypothesis that ATG proteins represent potentially useful biomarkers of PD because they reflect the disease severity and contribute to an early diagnosis.

Autophagy is a complex self-digestive process that removes aggregated proteins, damaged organelles, and intracellular pathogens. ^30^Beclin1 and ATG5 are two key ATGs involved in autophagosome biogenesis; thus, decreased expression of these proteins is responsible for impairments in the lysosomal autophagy pathway and is related to the pathological process of PD^20^. Additionally, the LC3B level in the CSF showed the most prominent difference between the PD and control groups in our study, and LC3B is a autophagy-specific cargo that is a notable marker of autophagosomes. However, increased numbers of autophagosomes are present in post-mortem brain tissues from patients with PD and cellular models of PD^21^. Thus, our results should be interpreted cautiously, and the disruptions in the lysosomal autophagy pathway might differ between early and advanced stages of PD pathogenesis. Furthermore, an interpretation of our results as showing decreased autophagy flux a subsequent decrease in autophaogosome formation that results in the accumulation of lysosomes in patients with early-stage PD contradicts our results that LAMP2 levels were decreased in the CSF from subjects with PD. Therefore, the lack of ATG proteins in the CSF should not be directly interpreted as indicating that a decrease in autophagy flux is involved in PD pathogenesis. Because lysosomal dysfunction might be an early event in PD^22^, and the LAMP2 level was decreased in our study, the decreased CSF levels of ATG proteins reported here might be caused by excessive trapping of autophagosomes in the brain tissue. Therefore, the significance of CSF levels of ATG proteins in PD pathogenesis remains unclear, and studies estimating the levels of ATG proteins in brain tissues and CSF are required to resolve this issue.

Interestingly, in our study, the LC3B level was significantly correlated with the severity of motor symptoms, dopamine transporter imaging data, and conventional CSF biomarkers, including α-synuclein and total tau. In previous studies, α-synuclein was correlated with Unified Parkinson’s Disease Rating Scale (UPDRS) part 3 score or Hoehn and Yahr (HY) stage, but these correlations were not found in many other studies^10,23^. Similarly, diverse results for the correlations between the total tau level or tau ratio with clinical severity have been reported, while the tau protein generally exhibits a distinct pattern associated with cognitive abnormalities in PD^24–26^. Therefore, based on our results, the LC3B level in the CSF represents a potentially useful marker of disease severity. Additionally, although the CSF levels of the total tau and phosphorylated tau proteins are reported to be associated with the presynaptic dopaminergic system based on findings from dopamine transporter imaging, no study has investigated the correlation between ATG proteins and dopaminergic imaging data^27^. In the present study, LC3B levels were significantly correlated with the AIs in the putamen and caudate nucleus. Patients with PD usually have asymmetric involvement, and asymmetric uptake by the dopamine transporter is a characteristic pattern observed in patients with PD^28^. Moreover, the AI has been used for the differential diagnosis of PD in previous studies^29,30^, and is also regarded as a marker of disease progression or the response to dopaminergic medications^31,32^. Therefore, the AI may be the most representative marker of disease progression in patients with PD, and an appropriate control for the laterality of parkinsonism in this study. Interestingly, the LC3B level exhibited a strong correlation with the AI, but not the standardized uptake value (SUV), of other specific anatomical structures in our study. Thus, an association between dysregulated autophagy and nigrostrital degeneration was observed at least in patients with early-stage PD, strengthening the usefulness of ATG proteins as potential biomarkers of early-stage PD.

Tau and α-synuclein exhibit close interactions. The aggregation of α-synuclein is accompanied by hyperphosphorylated tau, and α-synuclein facilitates the aggregation of the tau protein^33,34^. Considering the significant correlation between LC3B and α-synuclein in our study, ATG proteins and α-synuclein may interact. The activation of macroautophagy reduces the overexpression of the mutant α-synuclein protein, while inhibition of macroautophagy leads to a remarkable increase in the levels of endogenous or overexpressed α-synuclein in dopaminergic neurons. Conversely, α-synuclein itself may modulate the macroautophagy process, and transgenic mice overexpressing α-synuclein exhibited increased autophagosomes in previous studies^35^. However, α-synuclein might also affect chaperone-mediated autophagy, and the blockade autophagy flux and various molecular pathways affecting the lysosomal autophagy pathway are potential confounders in the interpretation of our results^36,37^. Furthermore, the significance of the α-synuclein level as a biomarker of PD remains controversial^38,39^. Therefore, the significance of the simple correlation between α-synuclein and LC3B levels in CSF remains unclear and further studies aiming to elucidate the precise significance of the interaction between these two proteins in the CSF are necessary.

Although a significant reduction in the total α-synuclein and total tau levels was observed in patients with PD, considerable overlap between the PD and control groups was also observed in our study. These findings are consistent with several previous studies, but one study examining drug-naïve patients with early-stage PD reported a significant reduction in the p-tau, total α-synuclein and total tau levels in patients with PD^8^. In addition, total and p-tau levels in the CSF correlated with dopamine transporter imaging data in only limited ROIs in our study, unlike a previous study^27^. Nevertheless, conflicting results have been reported for conventional CSF biomarkers, and the discrepancies among these studies might be due to differences in the sample size, diagnostic inaccuracy, the contributions of comorbid conditions, and a lack of standardization in the sampling protocol^24^.

Therefore, our results should be interpreted with caution, and further studies with strict protocols for data acquisition, CSF handling, and delicate adjustments of confounding factors are needed, although we confirmed that LC3B displayed a higher sensitivity and specificity for the diagnosis of PD compared to α-synuclein and total tau levels.

Our study has some important limitations. First, impairments in the lysosomal autophagy pathway are not a PD-specific mechanism and could be related to other neurodegenerative diseases. LAMP-1, LAMP-2, and LC3 levels are significantly increased in the CSF from patients with Alzheimer’s disease, and the autophagy biomarkers LC3B and Beclin1 correlate with clinical outcomes and disease severity in patients who experienced an acute stroke^40,41^. Although LC3B levels were markedly reduced in the PD group and strongly correlated with imaging data, clinical parameters and other CSF biomarkers in our study, the alteration in the LC3B level in the CSF was also observed in the two studies mentioned above. We adopted very strict inclusion criteria for patients with PD to minimize the possible confounding effects of comorbidities. Additionally, the PD-specific lysosomal autophagy pathway should be studied in the future. Second, ATG proteins, including LC3B, do not precisely reflect autophagy flux. A decreased LC3B level might not only be due to reduced autophagosome formation but also to increased autophagosome degradation. Furthermore, ATG proteins might be entrapped in autophagosomes due to the lack of lysosomal activity. CSF levels of autophagy marker proteins in patients with PD are likely affected by the dynamics of ATG proteins and may not reflect an actual change in the autophagy process. Therefore, the meaning of the decreased levels of ATG proteins in patients with PD remains unclear and an investigation of autophagy markers in brain specimens should be simultaneously conducted along with the monitoring of autophagy flux *in vivo* instead of simply measuring ATG protein levels to answer this question. In addition, the stability of these proteins in the CSF could be another concern. We tried to collect CSF samples under the same conditions, such as daytime collection from drug-naïve patients, as much as possible to minimize this limitation. Third, our results showed some discrepancies from previous studies, potentially due to the use of strict inclusion and exclusion criteria for enrolment to exclude possible confounding factors. Additionally, we enrolled drug-naïve patients with early-stage PD in the present study. Consequently, the sample size was relatively small and ATG proteins were not evaluated in patients with advanced PD. Finally, our study was designed as a cross-sectional study, and thus a longitudinal study with a larger population is required to determine the suitability of using CSF ATG proteins as biomarkers of PD progression.

Despite these limitations, our study examined a relatively homogenous PD group through the use of very strict inclusion and exclusion criteria to minimize possible confounding effects. We excluded subjects with white matter changes, comorbidities or medications that potentially affected levels of ATG proteins in the CSF. Furthermore, we also excluded subjects with any RBCs in their CSF to minimize contamination from RBCs, which can influence α-synuclein levels.

In conclusion, autophagy and lysosomes are involved in the pathogenesis of early-stage PD. Additionally, CSF ATG proteins, particularly LC3B, represents a potential biomarker for the diagnosis of early-stage PD. Moreover, LC3B levels in the CSF might also indicate disease severity in patients with early-stage PD.

## Methods

### Study population and clinical assessments

This study was approved by the Institutional Review Board of Gangneung Asan Hospital, and performed according to the principles of the Declaration of Helsinki. All enrolled subjects provided written informed consent. We prospectively recruited 32 drug-naïve patients with early-stage PD and 28 healthy controls at the Movement Disorders Clinic of Gangneung Asan Hospital from January to December, 2016. PD was diagnosed using the United Kingdom Parkinson’s Disease Society Brain Bank criteria, and FP-CIT PET. Parkinsonian motor symptoms were evaluated with the UPDRS part 3 and the HY stage, early-stage PD was defined as HY stage less than 3^42^. Non-motor symptoms were investigated in patients with PD using the Non-motor Symptoms Scale (NMSS)^43^. Additionally, Beck’s depression scale and the Korean version of Montreal Cognitive Assessment (MoCA-K) were performed to assess mood and cognitive symptoms in all enrolled subjects^44,45^. All clinical assessments were performed before patients were treated with dopaminergic medications. Basic demographic and laboratory data, including age, gender, haemoglobin level, haematocrit, and blood urea nitrogen, creatinine, aspartate aminotransferase, alanine aminotransferase levels were obtained in this study.

We excluded subjects 1) with abnormalities on the magnetic resonance imaging; 2) with a cognitive deficit (MoCA-K score less than 23); 3) who were taking medications known to regulate the autophagy pathway, including statins, carbamazepine, and vitamin D supplements; 4) who were diagnosed with other diseases, including cerebrovascular disease, diabetes, nervous or systemic infection, malignant diseases such as renal or hepatic failure, recent head trauma or surgery; and 5) whose CSF sample contained any number of red blood cell (RBCs) or white blood cell (WBCs) in a routine analysis.

### CSF collection and analysis

#### CSF sampling

CSF sampling and collection were performed according to a published protocol and guideline^46^. All CSF samples were collected between 9 and 12 a.m. after an overnight fasting from subjects with a drug-naïve status at admission to minimise interference from medications and lifestyle. CSF samples were collected in sterile, siliconized polypropylene tubes, and the first 2 mL of the CSF sample were sent to the laboratory for routine testing for cell counts, total protein levels, and glucose levels. An additional 15 mL of CSF was transferred to a 5-mL conical polypropylene tube at room temperature, mixed gently, centrifuged at 1500 g for 10 minutes at room temperature, and transferred to precooled 1.0-mL siliconized polypropylene aliquot tubes followed by immediate freezing on dry ice and storage in a −80 °C freezer. All CSF analyses were performed by a single laboratory technician who was blinded to the clinical data. All standards, aqueous controls, and CSF samples were analysed in duplicate in each run. The value of each result was defined as the arithmetic mean of the calculated concentration of duplicates.

#### Immunoassay

Concentrations of three PD biomarkers, total tau, tau phosphorylated at threonine 181 (p-tau) and α-synuclein, in the CSF were measured with commercially available Human Total tau, p-tau and α-synuclein ELISA Kits (Invitrogen, CA, USA) according to the manufacturer’s protocol. Briefly, 100 μL/well of diluted standards from reconstituted stocks and duplicate CSF samples (100 μL/well) were added to the capture antibody-coated plate and incubated at room temperature for 3 hours. 100 μL/well of biotinylated detection antibody were added, and the plate was incubated at room temperature for 2 hours. After the incubation, 100 μL/well of streptavidin horseradish peroxidase were added and incubated for 30 minutes at room temperature. After washing the plate four times, a mixture of 2 different chemiluminescent substrates was added and the optical density was measured using a Microplate reader (BioTek instruments, Inc., Winooski, VT, USA) at a wavelength of 450 nm. The proteins were quantified from standard curves using a linear regression analysis. Similarly, Beclin1, LAMP-2 and LC3B ELISA Kits (MYBioSource, San Diego, CA, USA) were used with a similar method to detect autophagy biomarkers. The coefficients of variation for all CSF markers in the laboratory are: α-synuclein (inter-assay: 7.9%, intra-assay: 7.2%); total tau (inter-assay: 9.9%, intra-assay: 5.9%); p-tau (inter-assay: 7.7%, intra-assay: 5.8%); LC3B (inter-assay: 10%, intra-assay: 8%); LAMP-2 (inter-assay: 12%, intra-assay: 10%).

### Immunoblot analysis

CSF samples were added to equal volumes of loading buffer (Invitrogen, CA, USA), and heated at 99 °C for 7 mins. Equal amounts of protein (15 μg) were loaded on an SDS-PAGE gel, electrophoretically separated, and transferred to polyvinylidene difluoride (PVDF) membranes. Membranes were blocked with 5% skim milk in Tris-buffered saline containing 0.1% Tween-20 (TBST) for 1 h and then incubated with primary antibodies (ATG5, ATG7 and p62 at a 1:1000 dilution; Cell Signaling Technology, Beverly, MA, USA) in TBST at 4 °C overnight. After three washes with TBST, membranes were incubated with the corresponding horseradish peroxidase-conjugated secondary antibodies (1:5000 dilution; Cell Signaling Technology) for 1 h at room temperature. Antibodies were detected using enhanced ECL Advance Western Blotting Detection Reagents (GE Healthcare, Buckinghamshire, UK) and LAS-4000 film (Fujifilm, Tokyo, Japan). Albumin (Sigma-Aldrich, St. Louis, MO, USA) served as a loading control.

### FP-CIT PET

FP-CIT PET scans were obtained 2 hours after the intravenous injection of an average of 185 MBq (5 mCi) of FP-CIT. All enrolled subjects underwent PET imaging in a Discovery ST8 scanner (GE Healthcare). The emission PET data were acquired for 15 minutes, and the computed tomography data were used for attenuation correction. The dopamine transporter binding state was evaluated through a semi-quantitative analysis of FP-CIT PET. The FP-CIT PET images were spatially normalized to the Montreal Neurological Institute (MNI) space using a standard FP-CIT PET template and the Statistical Parametric Mapping 8 (SPM8, http://www.fil.ion.ucl.ac.uk/spm) software implemented in MATLAB 12 for Windows (The MathWorks, Inc., Natick, MA, USA) as previously described^47^. Regional mean standardized uptake values (SUVmean) were extracted from the left and right caudate, the left and right putamen and the occipital cortex using volume-of-interest templates drawn in the MNI space^47^. The semi-quantitative analyses were performed using the SNBR and the AI.

The SNBRs of the putamen and caudate were calculated using the following equations:

(striatal SUVmean – occipital SUVmean)/occipital SUVmean

The AIs of the putamen and caudate were calculated as follows:

(higher striatal SNBR – lower striatal SNBR) / higher striatal SNBR.

### Statistical analysis

All data are presented as means ± standard deviations. All statistical analyses were performed with a commercial statistical software program (SPSS 18.0, Chicago, IL, USA). Categorical variables were compared using the chi-square test, and continuous variables with normally distributed data were compared using a standard t-test. The Mann-Whitney U test was used to compare variables that did not meet the assumption for a normal distribution. A receiver operating characteristic (ROC) curve analysis was performed using the ‘pROC’ package to determine whether specific proteins served as diagnostic biomarkers, and we used the ‘OptimalCutpoints’ package to calculate the optimal cut-off value for the difference between the control and PD groups by measuring Youden’s Index (R version 3.4.1). The relationships between the FP-CIT PET values and levels of ATG proteins, including LC3B, Beclin1, ATG5, and LAMP-2, in patients with PD were explored using partial Spearman’s correlation analyses adjusted for age. Correlations between autophagy biomarkers and conventional CSF markers, including total tau, p-tau and α-synuclein, were also evaluated using Pearson’s correlation test. A *p*-value* < *0.05 was considered statistically significant.

## Electronic supplementary material

Dataset 1
