# Supplementary material for: Cerebrospinal Fluid Levels of Autophagy-related Proteins Represent Potentially Novel Biomarkers of Early-Stage Parkinson’s Disease
Source: Sci Rep. 2018 Nov 15;8:16866. doi: 10.1038/s41598-018-35376-6 (PMC6237988; doi:10.1038/s41598-018-35376-6)
Supplement: Supplementary file 1 — Dataset 1 [file 41598_2018_35376_MOESM1_ESM.doc]

**Supplementary Information**

Supplementary Table

**Cerebrospinal Fluid Levels of Autophagy-related Proteins Represent Potentially Novel Biomarkers of Early-Stage Parkinson’s Disease**

Jinyoung Youn, Sang-Bin Lee, Hyo Sang Lee, Hyun Ok Yang, Jinse Park, Ji Sun Kim, Eungseok Oh, Suyeon Park, Wooyoung Jang

**Supplementary Table**

**Supplementary Table.** Partial analysis of the correlations between FP-CIT PET uptake values and CSF levels of α-synuclein, total tau and phosphorylated tau in patients with PD

|  | α-synuclein | | Total tau | | P-tau | |
| --- | --- | --- | --- | --- | --- | --- |
| **SNBR** | r | *p*-value | r | *p*-value | r | *p*-value |
| Right caudate | 0.04 | NS | 0.13 | NS | 0.30 | NS |
| Right putamen | 0.04 | NS | 0.12 | NS | 0.32 | NS |
| Left caudate | 0.34 | NS | 0.41 | **< 0.05** | 0.30 | NS |
| Left putamen | 0.37 | **< 0.05** | 0.22 | NS | 0.36 | **< 0.05** |
| AI (caudate) | -0.20 | NS | -0.28 | NS | -0.24 | NS |
| AI (putamen) | -0.27 | NS | -0.44 | **< 0.05** | -0.21 | NS |

FP-CIT PET: [^18^F] *N*-(3-fluoropropyl)-2β-carbon ethoxy-3β-(4-iodophenyl) nortropane PET; CSF: cerebrospinal fluid; PD: Parkinson’s disease; SNBR: specific to non-specific binding ratio; p-tau: phosphorylated tau; AI: asymmetry index.

Adjusted for age

Bold values indicate a statistically significant difference; NS: not significant

The asymmetry index was calculated as follows: (better uptake - worse uptake) / better uptake * 100.
